# Identification of Common Genes and Pathways in Eight Fibrosis Diseases

**DOI:** 10.3389/fgene.2020.627396

**Published:** 2021-01-15

**Authors:** Chang Gu, Xin Shi, Xuening Dang, Jiafei Chen, Chunji Chen, Yumei Chen, Xufeng Pan, Tao Huang

**Affiliations:** ^1^Department of Thoracic Surgery, Shanghai Chest Hospital, Shanghai Jiao Tong University, Shanghai, China; ^2^Department of Thoracic Surgery, Shanghai Pulmonary Hospital, Tongji University School of Medicine, Shanghai, China; ^3^Department of Cardiology, Shanghai Chest Hospital, Shanghai Jiao Tong University, Shanghai, China; ^4^Department of Colorectal and Anal Surgery, Xinhua Hospital, Shanghai Jiao Tong University School of Medicine, Shanghai, China; ^5^Shanghai Colorectal Cancer Research Center, Shanghai, China; ^6^Department of Nuclear Medicine, Ren Ji Hospital, Shanghai Jiao Tong University School of Medicine, Shanghai, China; ^7^Bio-Med Big Data Center, CAS Key Laboratory of Computational Biology, CAS-MPG Partner Institute for Computational Biology, Shanghai Institute of Nutrition and Health, Chinese Academy of Sciences, Shanghai, China

**Keywords:** fibrotic diseases, genes, pathways, Monte Carlo feature selection, CTGF

## Abstract

Acute and chronic inflammation often leads to fibrosis, which is also the common and final pathological outcome of chronic inflammatory diseases. To explore the common genes and pathogenic pathways among different fibrotic diseases, we collected all the reported genes of the eight fibrotic diseases: eye fibrosis, heart fibrosis, hepatic fibrosis, intestinal fibrosis, lung fibrosis, pancreas fibrosis, renal fibrosis, and skin fibrosis. We calculated the Kyoto Encyclopedia of Genes and Genomes (KEGG) and Gene Ontology (GO) enrichment scores of all fibrotic disease genes. Each gene was encoded using KEGG and GO enrichment scores, which reflected how much a gene can affect this function. For each fibrotic disease, by comparing the KEGG and GO enrichment scores between reported disease genes and other genes using the Monte Carlo feature selection (MCFS) method, the key KEGG and GO features were identified. We compared the gene overlaps among eight fibrotic diseases and connective tissue growth factor (CTGF) was finally identified as the common key molecule. The key KEGG and GO features of the eight fibrotic diseases were all screened by MCFS method. Moreover, we interestingly found overlaps of pathways between renal fibrosis and skin fibrosis, such as GO:1901890-positive regulation of cell junction assembly, as well as common regulatory genes, such as CTGF, which is the key molecule regulating fibrogenesis. We hope to offer a new insight into the cellular and molecular mechanisms underlying fibrosis and therefore help leading to the development of new drugs, which specifically delay or even improve the symptoms of fibrosis.

## Introduction

Acute and chronic inflammation often leads to fibrosis, which is also the common and final pathological outcome of chronic inflammatory diseases (Rockey et al., 2015). Fibrosis is defined as overaccumulation of fibrous connective tissue in and around the tissues with inflammation or damage, triggering irreversible scar formation. The clinical manifestations are renal disease, idiopathic pulmonary fibrosis (IPF), heart failure, end-stage liver diseases, and so on (Bataller and Brenner, 2005). Besides, fibrosis can also be observed in many chronic autoimmune diseases, such as rheumatoid arthritis, scleroderma, myelofibrosis, and Crohn disease. But the common characteristics of these fibrosis diseases were still unknown.

Fibrosis can affect chronic graft rejection, tumor invasion and metastasis, and the pathogenesis of many progressive myopathies. With regard to chronic graft rejection, fibrosis is one of the most common symptoms in chronic graft rejection. For example, liver transplantation in children has a 20-year survival of more than 80% at present, but the long-term results of these grafts still remain uncertain. Biopsies after liver transplantation show idiopathic post-transplant hepatitis and graft fibrosis occur even in children with good graft function (Kelly et al., 2016). As for tumor invasion and metastasis, carcinoma-associated fibroblasts are able to enhance tumor cells migration and invasion via activating the process of specific pathways. For example, as lung cancer maintains the leading cause of cancer-related deaths, IPF has been demonstrated that it increases the risk of lung cancer development by 7–20%, and there are multiple common molecular processes that associated IPF with lung cancer, such as epithelial–mesenchymal transition (EMT), endoplasmic reticulum stress, and abnormal expression of growth factors (Gu et al., 2018, 2020a,b; Ballester et al., 2019; Jiao and Yang, 2020). In the tissue of myopathies, there is prominent endomysial fibrosis, but little or no inflammation.

The fact that fibrotic changes are commonly observed in different diseases of diverse organ systems suggests common pathogenic pathways (Rockey et al., 2015). The wound healing in the fibrotic tissue is regulated by complex processes within different cells, and therefore some specific molecular pathways are activated. For example, in IPF, the fibrosis starts from the lung periphery to the lung center, finally causing respiratory failure. The underlying mechanisms of IPF were proven that elevated mechanical tension activates a transforming growth factor β (TGF-β) signaling loop in alveolar stem cells (AT2).

In this study, we proposed a new computational method incorporating feature engineering and feature selection algorithms to explore the common controlling genes and corresponding pathways among eight different organs’ fibrosis. The key genes and pathways were revealed, and the cross-talks between diseases were investigated. These results were helpful for understanding the molecular mechanisms of fibrosis diseases and finding new therapeutic indications of existing drugs, i.e., drug repositioning.

## Materials and Methods

### The Reported Genes of the Eight Fibrotic Diseases

All the genes of the related eight fibrotic diseases (eye fibrosis, heart fibrosis, hepatic fibrosis, intestinal fibrosis, lung fibrosis, pancreas fibrosis, renal fibrosis, and skin fibrosis) extracted from published researches are listed in Supplementary Table 1. In Supplementary Table 1, “1” refers to the genes associated with the specific fibrotic diseases, whereas “0” means the genes have no relationship with the specific fibrotic diseases. We compared the reported genes of the eight fibrotic diseases using R package SuperExactTest,^1^ which has the function of identification of sets of objects with shared features, which is a common operation in all disciplines. Analysis of intersections among multiple sets is fundamental for in-depth understanding of their complex relationships. This package implements a theoretical framework for efficient computation of statistical distributions of multiset intersections based on combinatorial theory and provides multiple scalable techniques for visualizing the intersection statistics (Wang et al., 2015). There were 954 genes that were associated with at least one of the eight fibrotic diseases. In each fibrotic disease, the numbers of reported genes are listed in Table 1.

**TABLE 1:** The number of reported genes in the eight fibrotic diseases.

Index	Disease	No. of reported genes
1	Eye fibrosis	39
2	Heart fibrosis	207
3	Hepatic fibrosis	173
4	Intestinal fibrosis	150
5	Lung fibrosis	185
6	Pancreas fibrosis	43
7	Renal fibrosis	160
8	Skin fibrosis	125

### Encoding the Fibrotic Disease Genes With KEGG and GO Features

We calculated the KEGG (Kyoto Encyclopedia of Genes and Genomes) and GO (Gene Ontology) enrichment scores of all fibrotic disease genes. For each specific fibrotic disease, the reported genes of this disease were considered as positive samples, and the other genes were considered as negative samples. The KEGG and GO enrichment scores (Shi et al., 2018; Gu et al., 2020c) were used as features to encode genes and characterize their functions.

The KEGG and GO enrichment scores were the functional profiles of a gene. To be more specific, we enriched the neighbors of genes in STRING network (version 11.0^2^) (Szklarczyk et al., 2018) on to KEGG pathway and GO terms. Given a gene *g*, let *S*(*g*) be a gene set consisting of genes that have functional associations with gene *g* in STRING network (Szklarczyk et al., 2018). Given a gene *g* and a GO term*GO*_*j*_, the GO enrichment score was defined as the −log_10_ of the hypergeometric test *P*-value (Chen et al., 2016) of the gene set *S*(*g*) and the GO term *GO*_*j*_, which can be computed as follows:

(1) $$S_{GO}\left(l,GO_{j}\right)=-\log_{10}\left(\sum_{k=m}^{n}\frac{\left(\begin{array}{c} M\\ m \end{array}\right)\left(\begin{array}{c} N-M\\ n-m \end{array}\right)}{\left(\begin{array}{c} N\\ n \end{array}\right)}\right)$$

where *N* was the total number of human genes in STRING database, *M* and *n* were the number of genes annotated to *GO*_*j*_ and the number of genes in *S*(*g*), respectively, and *m* was the number of genes in *S*(*g*) that were annotated to *GO*_*j*_.

Similarly, the KEGG enrichment scores can be calculated by replacing the GO terms with KEGG pathways. The higher enrichment score meant this gene can affect this biological function. In total, there were 22,130 features (324 KEGG enrichment scores and 21,806 GO enrichment scores). The GO (2019-Apr24) annotations were downloaded from ftp://ftp.geneontology.org/, and the KEGG (Release 91.0) annotations were extracted from https://www.kegg.jp/ using R/Bioconductor package KEGGREST^3^ on July 1, 2019.

### Identifying the Key KEGG and GO Features for Each Fibrotic Disease

The Monte Carlo feature selection (MCFS) method (Draminski et al., 2008) was applied to rank all the KEGG and GO features based on their importance in classification. It has been widely used and showed great power in identify robust key features for complex biological problems (Pan et al., 2018, 2020; Chen et al., 2020; Li et al., 2020a; Ren et al., 2020). As a supervised feature selection method, the MCFS method was based on tree classifiers. It constructed a series of tree classifiers on a series of subsets randomly selected from the whole dataset. By considering how much a feature contributed in these tree classifiers, the importance of this feature was calculated. By comparing with its importance calculated on permuted datasets, its significance can be calculated. As it ensembled a series of trees, the results were robust and trustworthy (Pan et al., 2019a,b,c, 2020; Li et al., 2020b).

For each fibrotic disease, the KEGG and GO enrichment features of the positive samples (the reported genes of this disease) and the negative samples (the other genes) were compared, and the relative importance (RI) of each feature was evaluated using MCFS algorithm. The significant KEGG and GO features were selected and analyzed. Software dmlab downloaded from http://www.ipipan.eu/staff/m.draminski/mcfs.html was used to apply the MCFS algorithm, and the default parameters were used.

## Results

### The Overlapped Genes of the Eight Fibrotic Diseases

**FIGURE 1:**
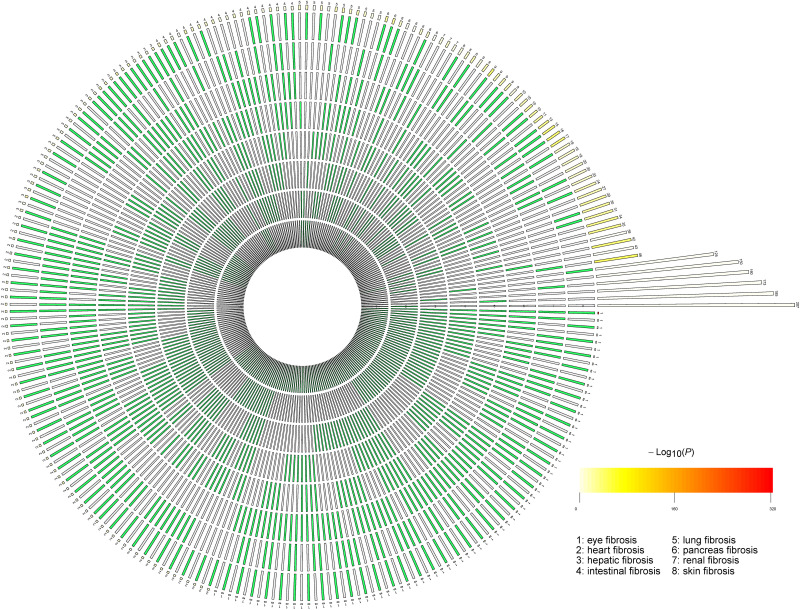
The number of overlapped genes among the eight fibrotic diseases. A circular plot illustrating all possible intersections and the corresponding statistics. The eight circles from inside to outside represent the eight fibrotic diseases (1, eye fibrosis; 2, heart fibrosis; 3, hepatic fibrosis; 4, intestinal fibrosis; 5, lung fibrosis; 6, pancreas fibrosis; 7, renal fibrosis; and 8, skin fibrosis), respectively. The height of the bars in the outer layer is proportional to the intersection sizes, as indicated by the numbers on the top of the bars. The color intensity of the bars represents the *P-*value significance of the intersections.

We compared the reported genes of the eight fibrotic diseases using R package SuperExactTest (see text footnote 1) (Wang et al., 2015). The results are shown in Supplementary Table 2. In Supplementary Table 2, degree 1 represents the original gene lists of the eight fibrotic diseases, degree 2 means the gene overlaps between any two groups, and degree 3 shows the gene overlaps among any three groups. By that analogy, degree 8 means the gene overlaps among all the eight groups. The data visualization is illustrated in Figure 1. The numbers of overlapped genes are listed over the histogram, and the darkness of the color represents how significant the overlap was. The connective tissue growth factor (CTGF) was finally identified as the common key molecule in the process of fibrosis.

### The Key KEGG and GO Features of the Eight Fibrotic Diseases

The key KEGG and GO features of the eight fibrotic diseases were screened by MCFS method. As shown in Supplementary Table 3, it means that if a gene could influence a specific function, it may cause a certain fibrotic disease.

As for eye fibrosis, the top three GO terms are GO:0033693 neurofilament bundle assembly, GO:1904530 negative regulation of actin filament binding, and GO:0031113 regulation of microtubule polymerization, respectively. GO:0033693 is associated with neurofilament bundle assembly, which means the assembly of neurofilaments into bundles, in which the filaments are longitudinally oriented, with numerous cross-bridges between them. GO:1904530 is related to negative regulation of actin filament binding, which means reducing physiological activities of actin filament binding. GO:0031113 is connected with the normal physiological activities of microtubule polymerization. Corneal fibrosis is the major type of eye fibrosis. Vimentin, a major structural type III intermediate filament, is a required component of keratocyte activation and differentiation corneal fibrosis, which often accelerates the process of fibrosis (Das et al., 2014).

As for heart fibrosis, the top three GO terms are GO:0032971 regulation of muscle filament sliding, GO:0070296 sarcoplasmic reticulum calcium ion transport, and GO:1990584 troponin complex, respectively. GO:0032971 is in connection with the process that regulates the frequency, rate, or extent of muscle filament sliding. GO:0070296 determines the movement of calcium ions, and GO:1990584 is associated with the cardiac troponin complex and influences muscle contraction. Therefore, muscle filament sliding and calcium ions have been proven to play important roles in the process of hypertrophic cardiomyopathy and heart fibrosis (Huang et al., 2014).

As for hepatic fibrosis, the top three GO terms are GO:0047747 cholate-CoA ligase activity, GO:0008508 bile acid:sodium symporter activity, and GO:0051264 mono-olein transacylation activity, respectively. GO:0047747 affects the activity of cholate-CoA ligase, which catalyzes some reactions in liver. GO:0008508 is related with bile acid and sodium ion transport. GO:0051264 is connected with mono-olein metabolism. Serum bile acids and total cholesterol (TC) are closely related to liver cirrhosis; the potential diagnostic value of total bile acid-to-cholesterol ratio (TBA/TC) for liver fibrosis has been proven (Yan et al., 2020).

As for intestinal fibrosis, the top three GO terms are GO:0032500 muramyl dipeptide binding, GO:0032498 detection of muramyl dipeptide, and GO:0045076 regulation of interleukin 2 (IL-2) biosynthetic process, respectively. GO:0032500 is related with muramyl dipeptide binding, whereas GO:0032498 is associated with detection of muramyl dipeptide. GO:0045076 regulates the process of IL-2 in fibrosis, which has also been proven in patients with cirrhosis and ascitic fluid (Juanola et al., 2016).

As for lung fibrosis, the top three GO terms are GO:0070950 regulation of neutrophil mediated killing of bacterium, GO:0070951 regulation of neutrophil mediated killing of Gram-negative bacterium, and GO:0004957 prostaglandin E receptor activity, respectively. GO:0070950 is related with regulation of neutrophil mediated killing of bacterium. GO:0070951 participates in regulation of neutrophil-mediated killing of Gram-negative bacterium. GO:0004957 means fibrogenesis via prostaglandin E receptor activity. It has been reported that neutrophil-mediated Gram-negative bacterial killing was connected with the cystic fibrosis (CF) lung (Vega-Carrascal et al., 2014).

As for pancreas fibrosis, the top three GO terms are GO:2000878-positive regulation of oligopeptide transport, GO:2000880-positive regulation of dipeptide transport, and GO:2001150-positive regulation of dipeptide transmembrane transport, respectively. All of the three are related to peptide transport. GO:2000878 is associated with positive regulation of oligopeptide transport, whereas GO:2000880 with positive regulation of dipeptide transport. GO:2001150 is related to positive regulation of dipeptide transmembrane transport. CF in the pancreas is characterized by an abnormality in cAMP-regulated chloride transport, which supports the findings of the predicted GO terms (Marino et al., 1991).

As for renal fibrosis, the top three GO terms are GO:0072015 glomerular visceral epithelial cell development, GO:0036057 slit diaphragm, and GO:0005362 low-affinity glucose:sodium symporter activity, respectively. GO:0072015 affects glomerular visceral epithelial cell development and therefore influences its formation to the mature structure. GO:0036057 associated a specialized cell–cell junction, which affects glomerular filtration. GO:0005362 is related to the transfer function of a solute. Renal fibrosis is often caused by renal glomerular sclerosis and interstitial fibrosis. Therefore, glomerular visceral epithelial cell development and formation, glomerular filtration, and transfer function act as the internal causes of renal fibrosis (Qi et al., 2020).

As for skin fibrosis, the top three GO terms are GO:0005600 collagen type XIII trimer, GO:0030936 transmembrane collagen trimer, and GO:0030316 osteoclast differentiation, respectively. GO:0005600 plays a role by collagen type XIII trimer, whereas GO:0030936 via transmembrane collagen trimer. Collagen trimer contributes to derangements in extracellular matrix (ECM) remodeling and leads to fibrosis (Madahar et al., 2018).

### The Cross-Talks Between Different Fibrotic Diseases

From the key KEGG and GO features of all the eight fibrotic diseases, we interestingly found overlaps of pathways within some specific fibrotic diseases. For example, renal fibrosis and skin fibrosis jointly influence GO:1901890-positive regulation of cell junction assembly. Some researchers have demonstrated that in renal fibrosis, MG132 successfully sustained cytoskeletal assembly and tight junction, preventing EMT process via RhoA-dependent TGF-β1 pathway, whereas in systemic sclerosis, endothelial junction–associated protein plays vital importance to the pathogenicity (Kanno et al., 2017).

**FIGURE 2:**
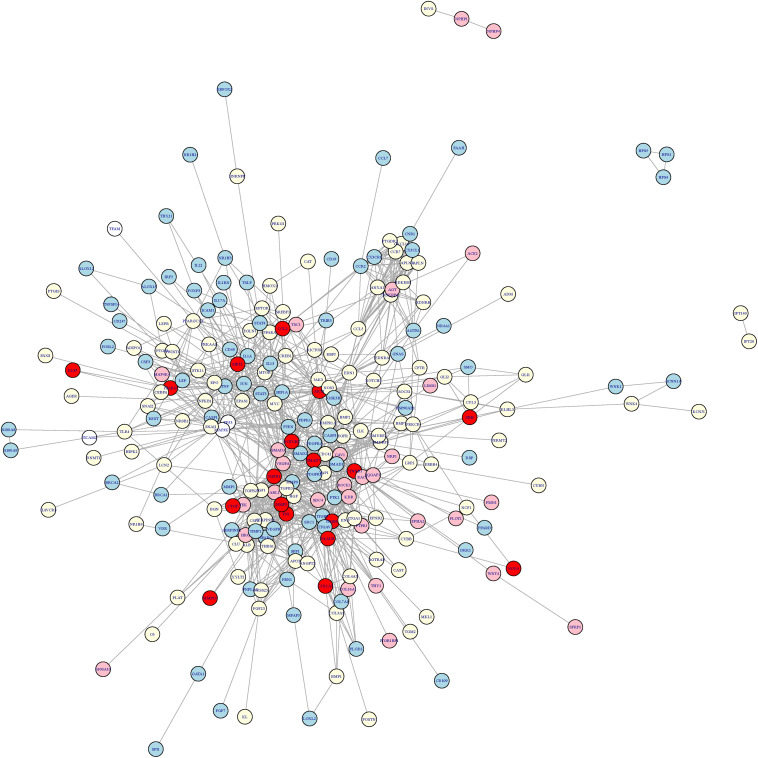
The cross-talk network between renal fibrosis and skin fibrosis. The genes in red refer to the overlaps between renal fibrosis and skin fibrosis, whereas the specific genes in renal fibrosis, skin fibrosis, and GO:1901890 are shown in light yellow, light blue, and pink circles, respectively. The overlapped genes between renal fibrosis and skin fibrosis included CCL2, SIRT1, KLF5, PPARG, AKT1, SHH, NOTCH, SMAD7, TGFB1, CTNNB1, MMP2, CTGF, FN1, ITGB1, PLAUR, MMP14, NOX4, and COL1A1.

To explore the cross-talk between renal fibrosis and skin fibrosis, we mapped the genes of renal fibrosis, the genes of skin fibrosis, and the genes of GO:1901890-positive regulation of cell junction assembly, which was the common GO feature between renal fibrosis and skin fibrosis, onto STRING network (Figure 2). In Figure 2, genes in red refer to the overlaps between renal fibrosis and skin fibrosis, whereas the specific genes in renal fibrosis, skin fibrosis, and GO:1901890 are shown in light yellow, light blue, and pink circles, respectively. As illustrated in Figure 2, the overlapped genes between renal fibrosis and skin fibrosis included CCL2, SIRT1, KLF5, PPARG, AKT1, SHH, NOTCH, SMAD7, TGFB1, CTNNB1, MMP2, CTGF, FN1, ITGB1, PLAUR, MMP14, NOX4, and COL1A1.

## Discussion

Fibrosis is a pathological characteristic of most chronic inflammatory diseases, and many deep learning methods have been developed to study human diseases (Wynn and Ramalingam, 2012; Chen Q. et al., 2019; Cheng and Ghany, 2020; Feng et al., 2020; Lan et al., 2020; Zhao et al., 2020). In recent years, fibrosis is recognized as a main reason of the occurrence of adverse events in many chronic inflammatory diseases. However, the underlying mechanisms in different organs are various and the generality among diverse fibrotic diseases still need to be uncovered. In this study, we applied a new computational method incorporating several machine learning algorithms to explore the common controlling genes and their corresponding pathways among eight different organs’ fibrosis.

### Common Genes

In our study, CTGF was identified as the common regulatory gene in the eight kinds of fibrotic diseases by MCFS method. It has been around 30 years since the discovery of CTGF from human umbilical vein endothelial cells. In previous researches, CTGF plays an important role in diverse diseases, including cancers, neurodegenerative diseases, systemic sclerosis, kidney diseases, pancreatic diseases, and so on, which means CTGF expresses generally. Mao et al. (2019) demonstrated that megakaryocytic leukemia 1 (MKL1) mediates TGF-β–induced CTGF transcription to promote renal fibrosis. CTGF knockdown dampened TGF-β–induced profibrogenic response in renal tubular epithelial cells. In cardiac fibrosis, Tan et al. (2019) developed an the lamin gene (LMNA) dilated cardiomyopathy (DCM) mouse model and found silencing of cardiac LMNA-induced DCM with associated cardiac fibrosis and inflammation and further uncovered that Yy1 suppresses DCM and cardiac fibrosis through regulation of bmp7 and CTGF. Besides, another study also proved that in patients with rheumatic heart disease, high CTGF expression was related to enlarged left atrial diameter, atrial fibrosis, and atrial anatomical remodeling (Chen J.Q. et al., 2019). In lung fibrosis, disintegrin and metalloproteinase 17, and CTGF were found to play critical roles in fibrotic procedures and contribute to lung fibrosis (Chen et al., 2018).

With regard to the gene overlaps of pathways within some specific fibrotic diseases, we have identified some common pathways and genes within renal fibrosis and skin fibrosis. For example, in chronic renal allograft injury resulting in progressive interstitial fibrosis, early urinary CCL2 is an independent predictor for the subsequent development of interstitial fibrosis and tubular atrophy at 24 months (Ho et al., 2010). Similarly, in systemic sclerosis (skin fibrosis), the levels of circulating CCL2, CCL3, and CCL5 chemokines were significantly higher in patients with systemic sclerosis than in controls.

### Common Pathways

Fibrosis and resultant organ failure result in approximately one-third of deaths worldwide (Zeisberg and Kalluri, 2013). Now that fibrosis is common and has harmful effects in almost all organs, it is a potential therapeutic target. As for predicted pathways, we have demonstrated some new pathways associated with the specific fibrotic diseases. In intestinal fibrosis, the GO term, GO:0045076, regulates the process of IL-2 in fibrosis. In patients with cirrhosis and ascitic fluid, Juanola et al. (2016) identified how the role of regulatory T cells played for compensating the inflammatory environment in cirrhosis when norfloxacin was applied, and they found norfloxacin immunomodulatory effect on IL-2 and interferon γ reduction. In lung fibrosis, GO:0070951 participates in regulation of neutrophil-mediated killing of Gram-negative bacterium. It has been reported that neutrophil-mediated Gram-negative bacterial killing was connected with the CF lung. The underlying mechanism was that galectin-9 (Gal-9) signaling through the T-cell Ig and mucin domain-containing molecule (TIM) and neutrophil TIM-3/Gal-9 signaling is perturbed in the CF airways due to proteolytic degradation of the receptor (Vega-Carrascal et al., 2014). GO:0004957 means fibrogenesis via prostaglandin E receptor activity. As Sieber et al. (2018) demonstrated, pathological features of pulmonary fibrosis include accumulation of myofibroblasts and increased ECM deposition in lung tissue; they developed a new assay with therapeutic potential in pulmonary fibrosis that acts via EP2 and EP4 receptors. In heart and renal fibrosis, angiotensin-converting enzyme inhibitors and angiotensin-receptor blockers that ameliorate cardiac and renal damage and fibrosis through many pathways such as TGF-β and SMAD pathways (Lambers Heerspink et al., 2013). In liver fibrosis, as hepatocytes process the ability of regeneration, intervention is needed for patients with hepatic fibrosis. For example, colchicine has been proven to prevent hepatic fibrosis via suppressing collagen secretion (Rockey, 2013). As the common pathways and genes were identified by our new computational method, old drugs for a specific fibrosis may be effective for another organ fibrosis.

## Conclusion

In conclusion, we identified that CTGF is acted as the key molecule regulating the processes of fibrogenesis and some common pathways within different fibrotic diseases via a new computational method. We hope to offer a new insight into the cellular and molecular mechanisms underlying fibrosis and therefore help lead to the development of new drugs that specifically delay or even improve the symptoms of fibrosis.

## Data Availability Statement

The original contributions presented in the study are included in the article/Supplementary Material, further inquiries can be directed to the corresponding author/s.

## Author Contributions

TH, XP, and YC: conception and design and administrative support. CG, XS, and XD: collection and assembly of the data and data analysis and interpretation. All authors wrote the manuscript and approved the submitted version.

## Conflict of Interest

The authors declare that the research was conducted in the absence of any commercial or financial relationships that could be construed as a potential conflict of interest.

## Footnotes

1 https://CRAN.R-project.org/package=SuperExactTest

2 https://string-db.org/

3 https://bioconductor.org/packages/KEGGREST/

## Supplementary Material

Supplementary Table 1 The reported genes of the eight fibrotic diseases.

Supplementary Table 2 The overlapped genes of the eight fibrotic diseases.

Supplementary Table 3 The key KEGG and GO features of the eight fibrotic diseases.
